# Stretchable Ag_2_Se Thermoelectric Fabric with Simple and Nonthermal Fabrication for Wearable Electronics

**DOI:** 10.1002/smsc.202400230

**Published:** 2024-09-01

**Authors:** Chaebeen Kwon, Sanghyeon Lee, Chihyeong Won, Kyu Hyoung Lee, Byeonggwan Kim, Sungjoon Cho, Taeyoon Lee

**Affiliations:** ^1^ Nanobio Device Laboratory School of Electrical and Electronic Engineering Yonsei University 50 Yonsei‐ro, Seodaemun‐gu Seoul 03722 Republic of Korea; ^2^ Andrew and Peggy Cherng Department of Medical Engineering Division of Engineering and Applied Science California Institute of Technology Pasadena CA 91125 USA; ^3^ Department of Materials Science and Engineering Yonsei University 50 Yonsei‐ro, Seodaemun‐gu Seoul 03722 Republic of Korea; ^4^ Department of Chemical Engineering and Applied Chemistry Chungnam National University 99 Daehak‐ro, Yuseong‐gu Daejeon 34134 Republic of Korea

**Keywords:** energy generator, pressure sensor, silver selenide, strain sensor, temperature sensor, thermoelectric fabric, wearable electronics

## Abstract

As the field of wearable electronics continues to expand, the integration of inorganic thermoelectric (TE) materials into fabrics has emerged as a promising development due to their excellent TE properties. However, conventional thermal methods for fabricating TE fabrics are unsuitable for wearable applications because of their high temperatures, resulting in rigid TE materials. Herein, a nonthermally fabricated silver selenide (Ag_2_Se) TE fabric is developed that can be effectively integrated into wearable applications. Ag_2_Se nanoparticles are densely formed within the fabric through a simple in situ chemical reduction process, resulting in remarkable electrical stability even after 10 000 cycles of mechanical deformation, such as stretching and compression. Notably, the fabricated Ag_2_Se TE fabric exhibits superior stretchability, stretching ≈1.36 times more than the thermally treated Ag_2_Se TE fabrics, while retaining its excellent electrical conductivity. Moreover, the TE unit exhibits 9.80 μW m^−1^ K^−2^ power factor, 134.45 S cm^−1^ electrical conductivity, and −26.98 μV K^−1^ Seebeck coefficient at 370 K. A haptic sensing glove based on the Ag_2_Se TE fabric as a sensor for detecting potential hazards is demonstrated. The glove effectively distinguishes between simple touch, physical pain, and high‐temperature hazards, ensuring user safety and prompt response.

## Graphical abstract

A nonthermally fabricated silver selenide (Ag_2_Se) thermoelectric (TE) fabric for wearable electronics is developed. This fabric maintains remarkable electrical stability and superior stretchability, outperforming thermally treated counterparts. The Ag_2_Se TE fabric, featuring a 9.80 μW m^−1^ K^−2^ power factor, 134.45 Scm^−1^ electrical conductivity, and −26.98 μV K^−1^ Seebeck coefficient, is demonstrated in a haptic sensing glove, effectively detecting touch, pain, and high‐temperature hazards.

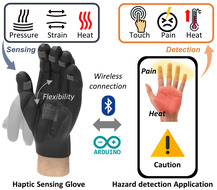

## 1 Introduction

With rapidly advancing textile‐based electronics, smart fibers have emerged as a critical component of functional textiles, distinguished by their remarkable responsiveness to various external stimuli.^[^ ^1^, ^2^, ^3^, ^4^, ^5^ ^]^ These smart fibers boast an extensive range of capabilities, including sensory functions^[^ ^6^, ^7^, ^8^, ^9^ ^]^ and energy‐harvesting abilities.^[^ ^10^, ^11^, ^12^ ^]^ This innovation is currently driving the emergence of novel fiber‐based wearable electronics, encompassing applications such as electronic skin,^[^ ^13^, ^14^ ^]^ health sensors,^[^ ^15^, ^16^ ^]^ and interfaces for human–machine interaction.^[^ ^17^, ^18^ ^]^ As the field of fiber‐based wearable electronics continues to evolve, the integration of thermoelectric (TE) materials into fibers has become a significant development.^[^ ^19^, ^20^, ^21^, ^22^, ^23^, ^24^ ^]^ TE materials efficiently convert temperature gradients into electrical output voltage and vice versa.^[^ ^25^ ^]^ This integration introduces a groundbreaking ability to harvest electrical energy from the natural heat generated by the human body.^[^ ^26^, ^27^ ^]^ Furthermore, TE fibers enable the detection of temperature variations and changes in posture by measuring output voltage and resistance.^[^ ^28^, ^29^, ^30^ ^]^

The pursuit of using inorganic TE materials for wearable applications has garnered significant attention in recent research.^[^ ^22^, ^23^, ^24^, ^31^, ^32^, ^33^ ^]^ Among these efforts, silver selenide (Ag_2_Se)‐based materials have emerged as promising candidate because of their unique TE properties.^[^ ^34^, ^35^, ^36^ ^]^ The incorporation of Ag_2_Se into fibers has been a cautious endeavor in several studies, primarily due to the inherent rigidity associated with inorganic TE materials.^[^ ^31^, ^32^, ^33^ ^]^ Yang et al. suggested an approach for creating a three‐dimensional (3D) TE generator with biaxial stretchability.^[^ ^31^ ^]^ By seamlessly integrating inorganic Ag_2_Se filmstrips into a knit fabric, aligned with the vertical heat flux direction, a stable temperature difference of 5.2 °C was realized when the fabric was in contact with the wrist at room temperature. Research is currently underway to explore TE units in fiber form, rather than in film form, to improve their suitability for wearable electronics applications. Vinodhini et al. reported a one‐step solvothermal method for integrating Ag_2_Se and Ag_2_S with conductive carbon fabric (CF).^[^ ^32^ ^]^ Ag_2_Se CF and Ag_2_S CF exhibited power factors (PFs) of 6.7 and 24 μW mK^−2^. However, during the material synthesis process, applying heat at 180 °C for 24 h frequently resulted in the formation of rigid structures. The only research on nonthermal Ag_2_Se fabric has been recently reported. Liu et al. introduced a two‐step impregnation method for fabricating a 3D TE network.^[^ ^33^ ^]^ The TE network demonstrated an elongation of over 100%. The resulting network‐based TE generator achieved an output power of 4 μW cm^−2^. Nevertheless, the process can be complicated and time‐consuming due to multiple steps, including solution preparation and impregnation. For the effective integration of Ag_2_Se into wearable electronics, a simple and nonthermal approach is essential.

Here, we fabricated a stretchable Ag_2_Se TE fabric through a simple and nonthermal process. Ag_2_Se nanoparticles (NPs) were densely and rapidly formed within the cotton fabric using a chemical reduction method without the need for thermal treatment. Due to the durable NP networks, the stretchable Ag_2_Se TE fabric exhibited remarkable electrical stability under repeated conditions of 20% lateral strain and 16 kPa normal pressure. Remarkably, it maintained its electrical path even under the strain of 200%, surpassing the thermally treated Ag_2_Se TE fabric in terms of electrical and mechanical performance. Moreover, the TE unit exhibited a PF of 9.80μW m^−1^ K^−2^, electrical conductivity of 134.45 S cm^−1^, and a Seebeck coefficient of −26.98 μV K^−1^ at 370 K. To demonstrate the practical utility of the fabricated fabric, a haptic sensing glove, capable of detecting changes in strain, pressure, and temperature, was developed, which effectively alerted users to potential hazards related to elevated temperatures and exhibited accurate sensing capabilities for pressure and tensile forces, thereby enhancing its real‐world applicability.

## 2 Results and Discussion

**Figure 1:**
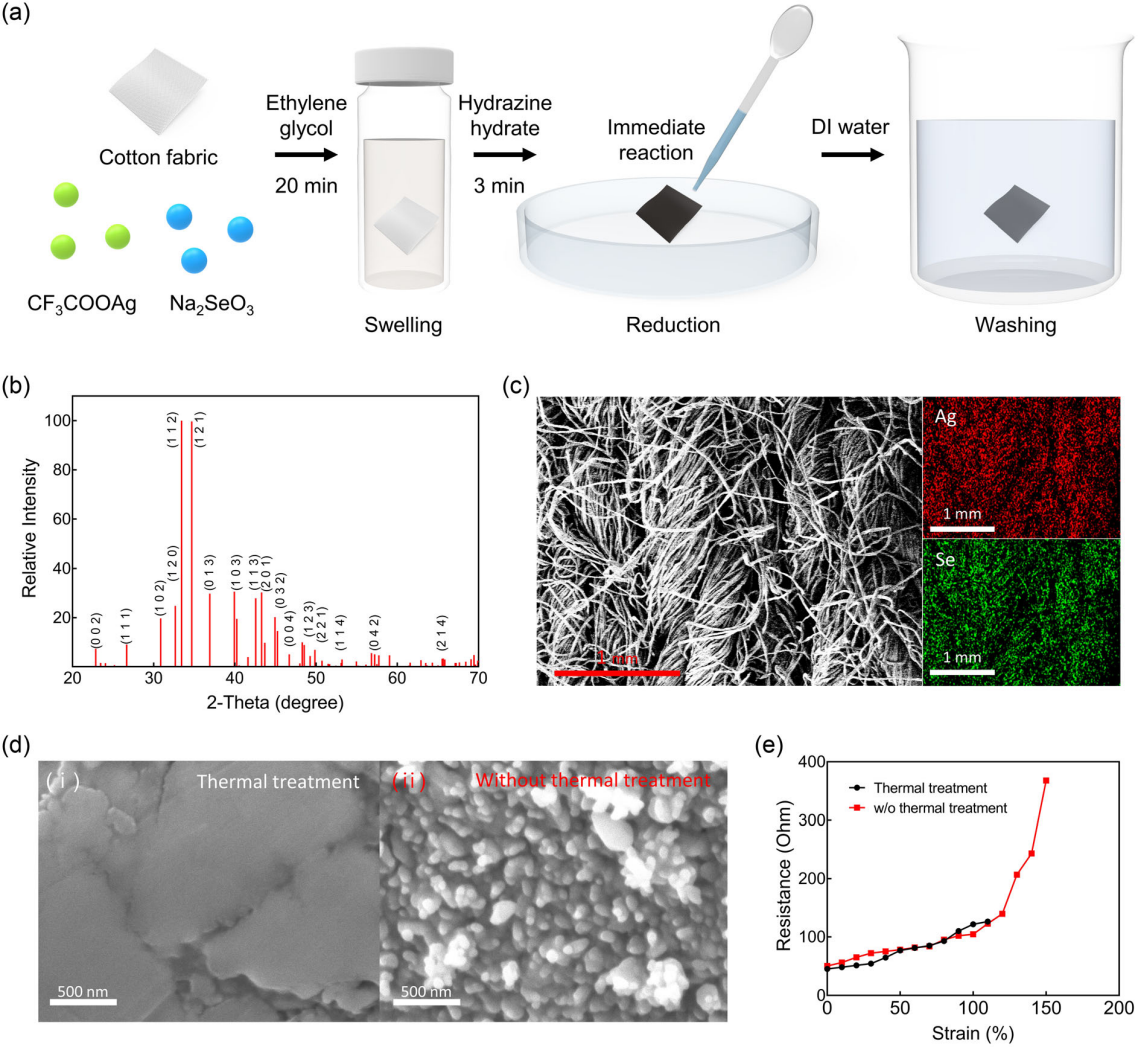
a) Schematic of the fabrication process of the Ag_2_Se TE fabric using the nonthermal chemical reduction method. b) XRD patterns of the Ag_2_Se TE fabric. c) Top view of SEM and EDS images of the Ag_2_Se TE fabric. d) (i) SEM image of Ag_2_Se TE fabric with thermal treatment. (ii) SEM image of Ag_2_Se TE fabric without thermal treatment. e) Resistance of the Ag_2_Se fabric with and without thermal treatment under different tensile strain levels.

The stretchable Ag_2_Se TE fabric is fabricated by incorporating Ag_2_Se NPs into a cotton fabric, as illustrated in **Figure** **1**a. This process was achieved using a simple and nonthermal chemical reduction method. To ensure the TE fabric stretchability, commercially available cotton fabric was used as the foundational structure. The nonthermal chemical reduction method comprises two distinct steps. Initially, the cotton fabric was immersed in the Ag_2_Se precursor solution for 20 min, allowing it to swell and absorb the solution. The Ag_2_Se precursor solution was formulated by blending two distinct precursor powders with an ethylene glycol (C_2_H_4_(OH)_2_) solvent. Specifically, silver trifluoroacetate (CF_3_COOAg) and sodium selenite pentahydrate (Na_2_SeO_3_) were selected as Ag and Se precursor. Subsequently, hydrazine hydrate (N_2_H_4_) was applied to the fabric to reduce the absorbed Ag_2_Se precursor solution, resulting in the rapid formation of dense Ag_2_Se NPs within the fabric. The reduction reaction between the precursor solution and the reductant occurred instantly without any thermal treatment. Following the reduction process, the fabric is washed with deionized (DI) water to remove any residual hydrazine hydrate. Figure 1b shows the X‐ray diffraction (XRD) patterns of the Ag_2_Se TE powder synthesized using the nonthermal chemical reduction method. These patterns revealed the crystallinity of Ag_2_Se and were aligned with the diffraction lines of (0 0 2), (1 1 1), (1 0 2), (1 2 0), (1 1 2), (1 2 1), (0 1 3), (1 0 3), (1 1 3), (0 3 2), (0 0 4), (1 2 3), (2 2 1), (1 1 4), and (0 4 2).^[^ ^34^, ^37^ ^]^ Figure S1, Supporting Information, shows an optical image of the Ag_2_Se TE fabric with a deep brown color. Figure 1c shows the top‐view images acquired through scanning electron microscopy (SEM) and energy‐dispersive X‐ray spectroscopy (EDS) for the Ag_2_Se TE fabric. The SEM image provided a detailed depiction of the structure of the TE fabric with a bundle of fine fibers. The stretchability of the TE fabric can be attributed to its interwoven fiber‐based structure. In EDS images, the formation of NPs was depicted in color, with Ag and Se NPs appearing as red and green dots. Figure 1d shows SEM images of the Ag_2_Se TE fabric with and without thermal treatment. Figure 1d‐(i) shows the surface of the Ag_2_Se TE fabric treated at 200 °C for 2 h. The surface became film‐like, making it susceptible to mechanical deformation caused by routine human activities, potentially resulting in the formation of cracks on the surface and potential loss of electrical conductivity. The film‐like Ag_2_Se layer can be easily peeled off after cyclic mechanical deformation (Figure S2, Supporting Information). Conversely, the surface of the nonthermally treated Ag_2_Se TE fabric maintained an NP morphology (Figure 1d‐(ii)). This NP morphology allowed external forces to disperse between NPs, making the fabric resistant to mechanical deformation and enhancing its suitability for wearable applications. The Ag_2_Se NPs remained attached to the surface of the fiber even after cyclic mechanical deformation (Figure S3, Supporting Information). Figure 1e shows the resistance of the Ag_2_Se TE fabric with and without thermal treatment under tensile strain. The Ag_2_Se TE fabric without thermal treatment maintained electrical conductivity over a broader range of tensile strain, stretching ≈1.36 times more than the thermally treated Ag_2_Se TE fabric. Notably, the Ag_2_Se TE fabric exhibited high electrical conductivity even without thermal treatment.

**Figure 2:**
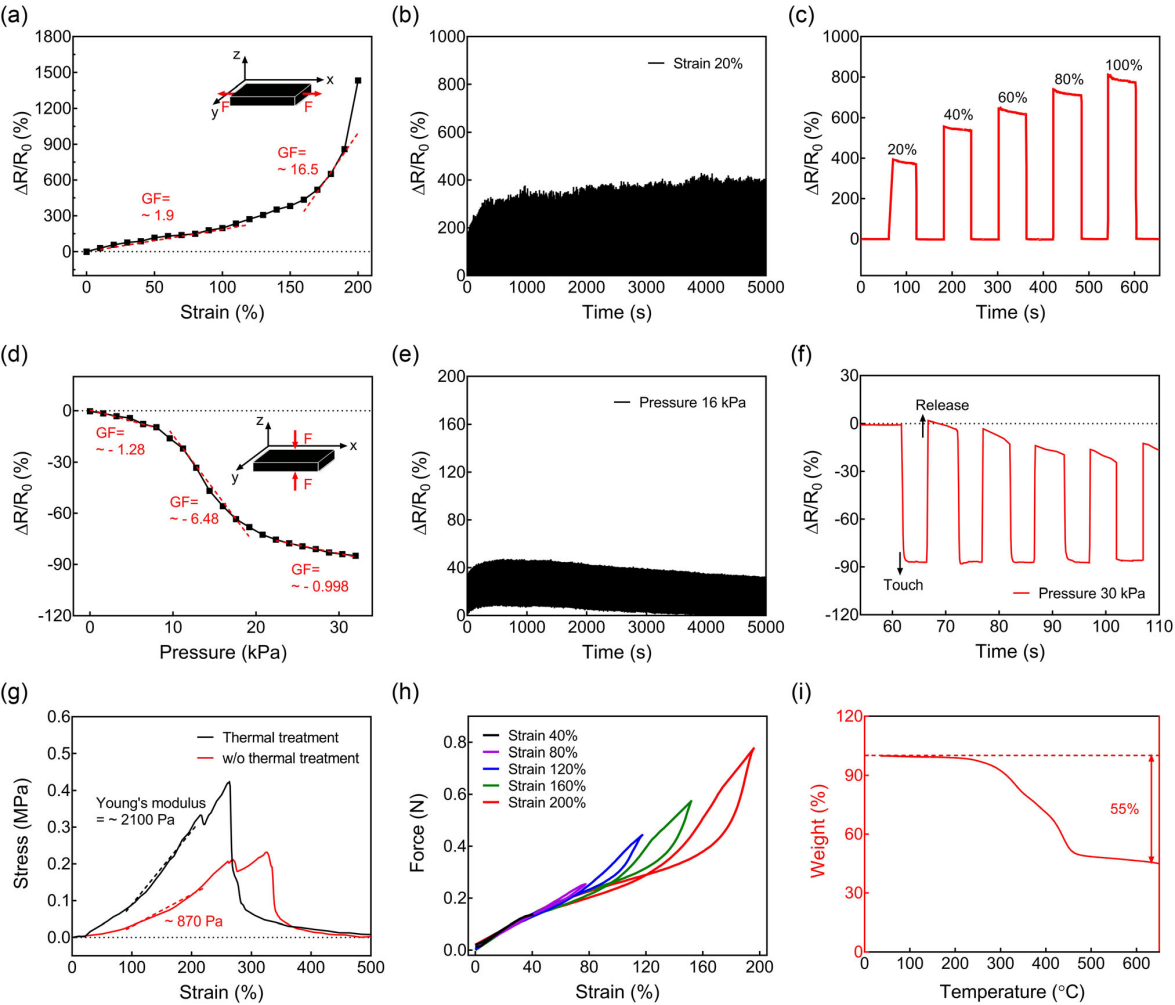
a) Relative changes in the resistance of the Ag_2_Se TE fabric under tensile strain levels up to 200%. b) Resistance changes of the Ag_2_Se TE fabric during 10 000 stretching cycles from 0% to 20% tensile strain. c) Relative changes in the resistance of the Ag_2_Se TE fabric during cyclic stretching and releasing deformation at a 20%, 40%, 60%, 80%, and 100% tensile strain. d) Relative changes in the resistance of the Ag_2_Se TE fabric under pressure up to 30 kPa. e) Resistance change of the Ag_2_Se TE fabric during 10 000 compressing cycles from 0 to 16 kPa. f) Relative changes in the resistance of the Ag_2_Se TE fabric during cyclic compression and release deformation at a pressure of 30 kPa for five cycles. g) Strain–stress curve of the Ag_2_Se TE fabric with and without thermal treatment up to mechanical fracture. h) Force of the Ag_2_Se TE fabric against tensile strain every 40% up to 200%. i) Weight (%) of Ag_2_Se NPs in the TE fabric determined by TGA.

**Figure** **2**a shows the relative changes in resistance according to the tensile strain of the Ag_2_Se TE fabric. As the applied tensile strain increased, the relative changes in resistance increased due to the intrinsic piezoresistance of Ag_2_Se NPs. This increase was reflected in the change in the gauge factor of the Ag_2_Se TE fabric, increasing from 1.9 to 16.5. The electrical reliability of the Ag_2_Se TE fabric was evaluated under repeated mechanical deformations. As shown in Figure 2b, a stretching test with 20% tensile strain was conducted. Remarkably, the Ag_2_Se TE fabric maintained high electrical reliability throughout 10 000 stretching cycles. Figure 2c shows the relative changes in the resistance of the Ag_2_Se TE fabric under stretching and releasing deformations at 20%, 40%, 60%, 80%, and 100% tensile strains. The increase in tensile strain corresponded to a proportional increase in the relative changes in resistance, demonstrating the ability to distinguish between different levels of tensile strain. Figure 2d shows the relative changes in resistance according to the pressure of the Ag_2_Se TE fabric. The relative changes in resistance decreased, reaching ≈−90% at the maximum compression point of 32 kPa due to the increased contact area between Ag_2_Se TE fibers under pressure, thereby improving electrical conductivity. The electrical stability of the Ag_2_Se TE fabric was confirmed under 16 kPa pressure. Figure 2e shows that even after 10 000 repeated compression cycles, the Ag_2_Se TE fabric maintained its electrical reliability. Figure 2f shows the relative changes in resistance observed in the Ag_2_Se TE fabric during cyclic compression and release deformations at 30 kPa pressure over five cycles. These cycles consistently exhibited a substantial reduction in relative changes in resistance, reaching ≈−90% when the Ag_2_Se TE fabric was touched. Under tensile strain, the Ag_2_Se TE fabric exhibited an increase in resistance, while pressure decreased the resistance. This distinct electrical behavior of the fabric depicted a clear differentiation between tensile strain and pressure. Figure 2g shows the strain–stress curve of the Ag_2_Se TE fabric with and without thermal treatment up to mechanical fracture. The Ag_2_Se TE fabric without thermal treatment fractured at 325% tensile strain, exhibiting Young's modulus of 870 Pa. However, after thermal treatment, the fabric exhibited a lower fracture point at 260% tensile strain, along with an increased Young's modulus of 2100 Pa, indicating that the nonthermally treated fabric was more flexible and stretchable than the thermally treated fabric. Figure 2h shows the force of the Ag_2_Se TE fabric as a function of tensile strain, 50–200% values. Mechanical hysteresis is in all loading and unloading cycles; however, its impact is negligible, emphasizing the mechanical stability of the fabric. Figure 2i shows the results of the thermogravimetric analysis (TGA) conducted on the Ag_2_Se TE fabric, revealing information about the weight percentages of Ag_2_Se NPs. The TGA graph indicated that Ag_2_Se NPs were densely formed within the TE fabric, constituting ≈45 wt%.

**Figure 3:**
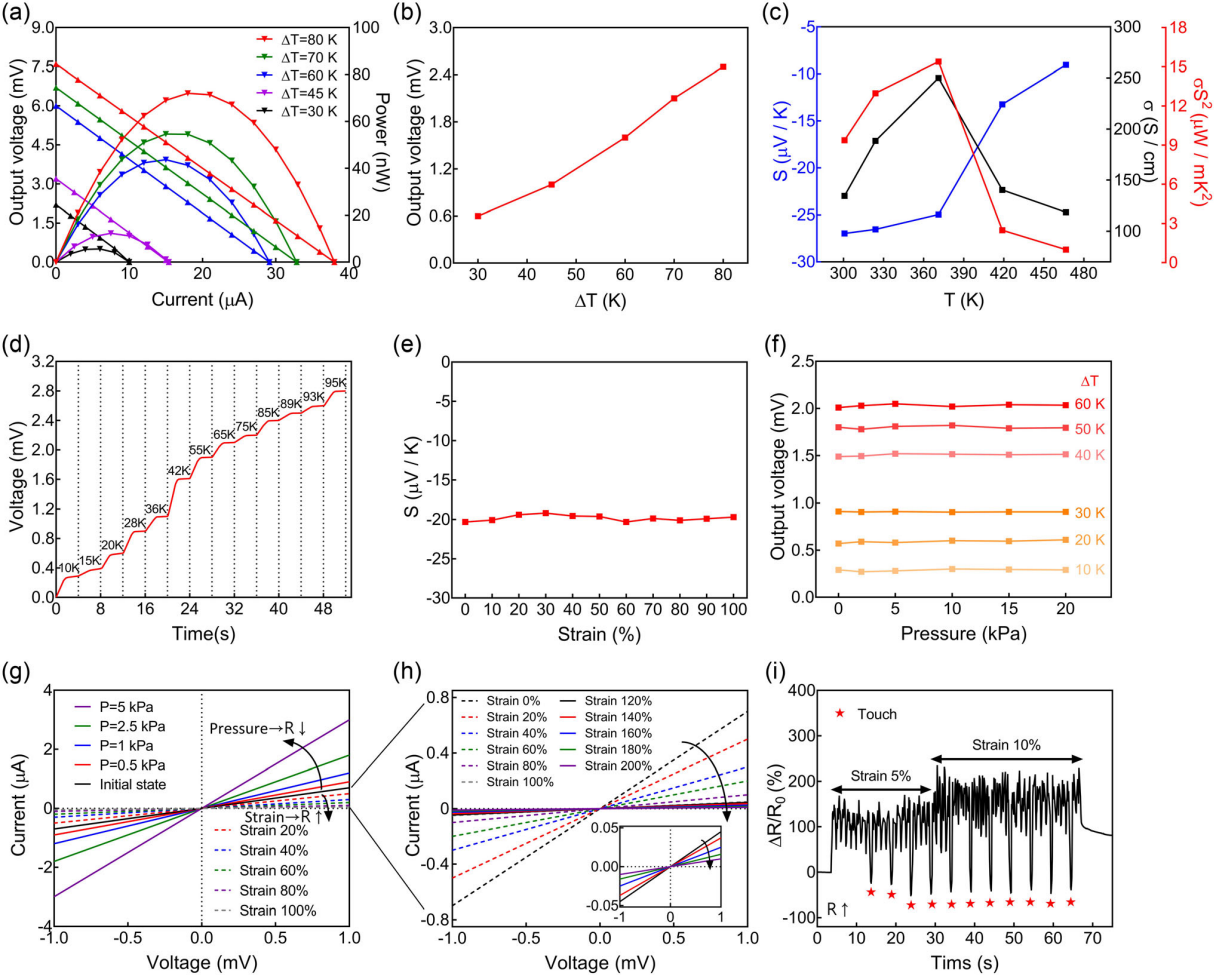
a) Output voltage and power of the Ag_2_Se TE fabric with current at various temperature differences (Δ*T*). b) Experimental output voltages of the Ag_2_Se TE fabric at various Δ*T* values. c) Temperature dependence of Seebeck coefficient (*S*), electrical conductivity (*σ*), and PF of the Ag_2_Se TE fabric. d) Sequential electrical responses of the Ag_2_Se TE fabric at various Δ*T*, ranging from 10 K to 95 K. e) S of the Ag_2_Se TE fabric under varying tensile strain up to 100%. f) Output voltages of the Ag_2_Se TE fabric under different pressures (0–20 kPa) at each Δ*T*, ranging from 10 K to 60 K. g) *I*–*V* curves of the Ag_2_Se TE fabric taken at different pressures (0–5 kPa) and tensile strain (0–100%). h) I–V curves of the Ag_2_Se TE fabric taken at different tensile strains, ranging from 0% to 200%. i) Relative changes in the resistance of the Ag_2_Se TE fabric under finger touch and repeated 5% and 10% tensile strain.

**Figure** **3**a–c shows the thermoelectrical performance of the Ag_2_Se TE fabric (5 × 1 × 10 mm^3^). Figure 3a shows the output performance with the current of the fabric at various temperature differences (Δ*T*). The maximum power output was determined by achieving a balance between external and internal resistances.^[^ ^38^, ^39^ ^]^ The maximum power values were ≈0.01656, ≈0.05376, ≈0.19392, ≈0.5250, and ≈0.082236 μW at various Δ*T* values of 30, 45, 60, 70, and 80 K, respectively. Figure 3b shows the experimentally measured output voltage of the Ag_2_Se TE fabric at several Δ*T* values, showing a linear increase as Δ*T* increases. Figure 3c shows the temperature‐dependent TE properties of the Ag_2_Se TE fabric, including the Seebeck coefficient (*S*), electrical conductivity (*σ*), and PF. Electrical conductivity, represented by the black line, increased but then sharply decreased from 250 to 140.36 S cm^−1^ as the temperature increased from 370 to 420 K. The blue line indicates the Seebeck coefficient with a negative sign, indicating that electrons predominate as the majority carriers in this n‐type material. The absolute Seebeck coefficient gradually decreased and then rapidly decreased from 24.97 to 13.24 μV K^−1^ within 370–420 K. Consequently, the PF increased from 9.79 μW m^−1^ K^−2^ at 300 K to a maximum of 15.58 μW m^−1^ K^−2^ at 370 K, followed by a sharp decrease when the temperature exceeded 370 K. Above 300 K, intrinsic conduction prevailed, resulting in enhanced thermal excitation and subsequent growth in the carrier concentration, and contributing to enhanced electrical conductivity. The abrupt change in TE performance from 370 to 420 K can be attributed to the phase transition of Ag_2_Se, transitioning from semiconducting orthorhombic to superionic cubic, occurring at ≈407 K.^[^ ^40^, ^41^ ^]^ Figure 3d shows the consecutive electrical responses of the Ag_2_Se TE fabric across various temperature gradients (10–95 K). The Ag_2_Se TE fabric efficiently transformed the thermal energy into electrical energy, yielding an output voltage. The peak output voltage, observed under the largest temperature difference of 95 K, reached ≈2.7 mV. Figure 3e shows the Seebeck coefficients of the Ag_2_Se TE fabric under various applied tensile strains. Notably, the Seebeck coefficients remain constant. As the Seebeck coefficient directly correlated with the output voltage, a parameter crucial for Δ*T* sensing, it indicated that the Δ*T* sensing ability of the fabric remained consistent under different tensile strain levels. Figure 3f shows that the output voltage of the Ag_2_Se TE fabric remained consistently stable across varying pressures (0–20 kPa) for each Δ*T* value, indicating that Δ*T* can be reliably detected using the fabric, even when it is subjected to applied pressure. Figure 3g shows current–voltage (*I*–*V*) curves under different applied pressures and tensile strains, showing the pressure and tensile strain sensing mechanism of the Ag_2_Se TE fabric. The curve slopes increased around the origin with increasing pressure levels due to the decreasing resistance of the fabric under applied pressure. Specifically, the resistance of the fabric decreases from 1.43 kΩ under 0 kPa to 1.11, 0.83, 0.56, and 0.33 kΩ under 0.5, 1, 2.5, and 5 kPa. Conversely, slopes exhibited a decrease around the origin with increasing tensile strain levels, as shown in Figure 3g,h. This decrease in slope was due to the increasing resistance of the fabric under the applied tensile strain. Figure 3h provides a closer look at the *I*–*V* curves from Figure 3g under different tensile strain levels. The resistance of the Ag_2_Se TE fabric was 1.43 kΩ under strain of 0% and increased to 2, 3.33, 5, 10, 20, 22.2, 27.03, 40, 62.5, and 100 kΩ under strain of 20%, 40%, 60%, 80%, 100%, 120%, 140%, 160%, 180%, and 200%, respectively. Due to these distinctive resistance behaviors under pressure and tensile strain, these two mechanical stresses were distinguished using the Ag_2_Se TE fabric. Consequently, the Ag_2_Se TE fabric enabled the ability to distinguish touch that occurred during repeated 5% and 10% tensile strains as shown in Figure 3i. Notably, when the fabric was touched, the relative changes in resistance decreased to ≈−40%. This contrasts starkly with the resistance changes of ≈150% and 200% observed during cyclic 5% and 10% tensile strain.

**Figure 4:**
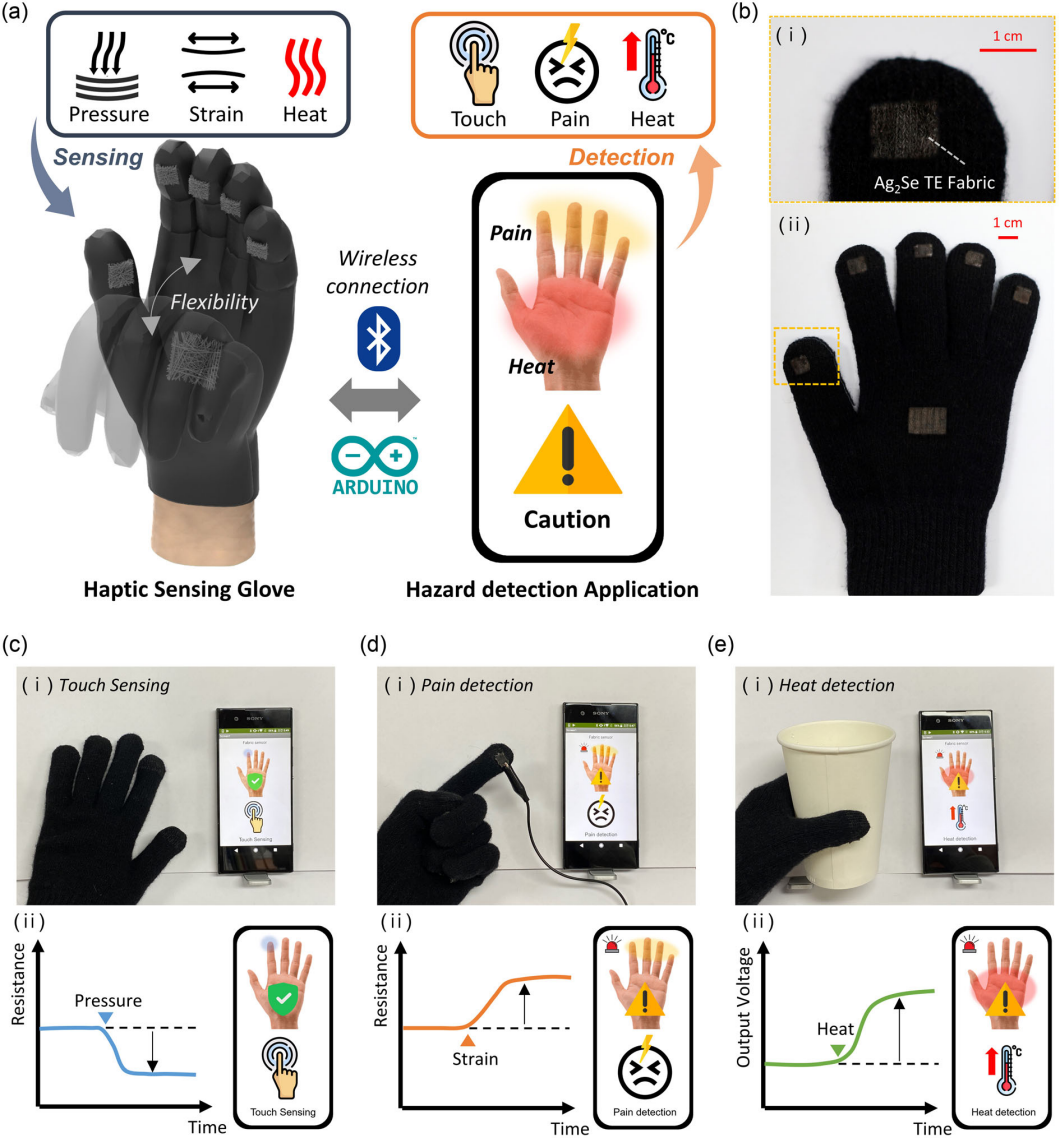
a) Schematic illustration of the haptic sensing glove‐based potential hazard detection system. b) Optical image of the haptic sensing glove. c) (i) Demonstration of touch sensing using the haptic sensing glove. (ii) Pressure sensing‐based touch sensing mechanism. d) (i) Demonstration of pain detection using the haptic sensing glove. (ii) Strain sensing‐based pain detection mechanism. e) (i) Demonstration of heat detection using the haptic sensing glove. (ii) Output voltage sensing‐based heat detection mechanism.

The Ag_2_Se TE fabric exhibited electrical responsiveness to pressure, strain, and temperature differences through changes in resistance and output voltage. We used these features to detect potential hazards such as physical pain and high temperatures using a glove integrated with Ag_2_Se TE fabrics as a haptic sensor. The haptic sensing glove was fabricated by simply weaving Ag_2_Se TE fabrics onto the fingertips and palms of a knitted glove. **Figure** **4**a shows a schematic of the hazard detection system using the fabricated haptic sensing glove. The haptic sensing glove transmits analog signals such as resistance and output voltage in response to pressure, strain, and heat generated during contact with Arduino. These signals are then transmitted to a mobile phone via a Bluetooth module. The application analyzed the received information about pressure, strain, and heat to determine whether it was a simple touch, physical pain, or high‐temperature hazard. The results were then displayed within the application accordingly. Figure 4b shows optical images of the haptic sensing glove, showing the Ag_2_Se TE fabrics embedded into the fingertips (1 × 0.7 cm^2^) and palm (2 × 1.2 cm^2^) of the knitted glove (Figure 4b‐(ii)). Figure 4b‐(i) shows a magnified view of the fabric structure at the fingertip of the haptic sensing glove. By integrating Ag_2_Se TE fabrics as sensing components, the haptic sensing glove exhibited great potential as a wearable electronic device, offering exceptional conformability, flexibility, and stretchability. Figure 4c–e shows the practical implementation of the haptic sensing glove for detecting simple touch, physical pain, and high‐temperature hazards. Figure 4c shows a demonstration of touch sensing detection, where glove touch interactions lead to a noticeable decrease in the resistance of the Ag_2_Se TE fabric, enabling the detection of simple touch inputs. Figure 4d shows pain detection, where the application of stimuli such as pinching induces tensile strain in the Ag_2_Se TE fabric, increasing its resistance and allowing the detection of physical pain through changes in fabric resistance. Figure 4e shows heat detection, where the temperature difference caused by holding hot objects triggered the generation of an output voltage in the Ag_2_Se TE fabric. This distinctive characteristic has enabled the detection of potential high‐temperature hazards. The applications of the haptic sensing glove, including the detection of simple touch, physical pain, and high‐temperature hazards, are presented in Movie S1 (Supporting Information). Additionally, the Ag_2_Se TE fabric demonstrates superior stretchability and a straightforward, nonthermal fabrication process compared to previously reported TE fibers and fabrics, as detailed in Table S1 (Supporting Information).

## 3 Conclusion

In this study, we successfully fabricated a stretchable Ag_2_Se TE fabric through a simple in situ reduction process without thermal treatment. This nonthermal fabrication method resulted in the formation of durable Ag_2_Se TE NP networks within the fabric, providing high stretchability up to 350% tensile strain. The Ag_2_Se TE fabric exhibited remarkable electrical reliability under 10 000 cycles of mechanical deformation such as 20% tensile strain and 16 kPa pressure, making it suitable for use as a wearable sensor. Moreover, the Ag_2_Se TE fabric achieved a PF of 15.58 μW m^−1^ K^−2^, electrical conductivity of 250 S cm^−1^, and a Seebeck coefficient of −24.97 μV K^−1^ at 370 K. The Ag_2_Se TE fabric demonstrated the capacity to generate different output voltages in response to various temperature differences based on the Seebeck effect. To demonstrate its practical application, we integrated the Ag_2_Se TE fabric into a knitted glove, resulting in the development of a haptic sensing glove. This glove was designed to detect various stimuli, such as touch, physical pain, and high‐temperature hazards, achieved by utilizing analog input signals derived from the resistance and output voltage of the Ag_2_Se TE fabric, influenced by pressure, strain, and temperature differences. This facile and nonthermally created Ag_2_Se TE fabric‐based electronic device can pave the way for the application of inorganic TE materials in a stretchable form in wearable devices.

## 4 Experimental Section

4.1

4.1.1

##### Fabrication of Ag_2_Se TE Fabric

Commercially available cotton fabrics were purchased and used as the foundation for the stretchable TE fabric. To create the Ag_2_Se precursor solution, a combination of 20 wt% silver trifluoroacetate (CF_3_COOAg) and 10 wt% sodium selenite (Na_2_SeO_3_) was mixed in an ethylene glycol (C_2_H_4_(OH)_2_) solvent. The cotton fabrics were then immersed in this solution for 20 min. Ag_2_Se NPs were obtained through in situ chemical reduction using hydrazine hydrate (N_2_H_4_) as a reducing agent (3 min, RT). After reducing the Ag_2_Se precursor, the fabrics were rinsed with DI water. This step was conducted multiple times to ensure the complete removal of any residual reductant. After the rinsing procedure, the fabrics were left to dry.

##### Experiments with a Haptic Sensing System

The haptic sensing glove was used as a pressure, strain, and temperature difference sensor to detect touch, physical pain, and high‐temperature hazards. Analog signals from the haptic sensing glove were received using Arduino Mega 2560. The Arduino Mega 2560 communicated with a smartphone via Bluetooth HC‐06. The Android application was developed using MIT App Inventor. Analog signals were transmitted to the App Inventor system and transformed into images alerting the user to touch, physical pain, and high‐temperature hazards.

##### Characterization

The top‐view and EDS images of the Ag_2_Se TE fabric were obtained by field‐emission SEM (FE‐SEM) (JSM‐IT500HR, JEOL). The surface images of the Ag_2_Se TE fabric with and without thermal treatment were also examined by FE‐SEM (JSM‐IT500HR, JEOL). The weight fraction of Ag_2_Se NPs in the fabrics was determined using a thermal analyzer (Q50, TA Instruments). The electrical and mechanical properties of the fabrics were evaluated using a source meter (B2901A, Keysight Technologies) and a force transducer (S2M, HBM), respectively. The stretching test of the Ag_2_Se TE fabric was conducted using a customized machine equipped with a motorized x‐axis stage (X‐translation stage, Jaeil Optical Corp). The compressing test of the Ag_2_Se TE fabric was examined using a customized pressure machine. The TE properties (electrical conductivity, Seebeck coefficient, and PF) of the Ag_2_Se TE fabric were analyzed using a four‐probe system (ZEM‐3, ULVAC‐RIKO). The output voltages of the Ag_2_Se TE fabric were measured using a custom‐made machine.

## Conflict of Interest

The authors declare no conflict of interest.

## Author Contributions

**Chaebeen Kwon**: Conceptualization (lead); Data curation (lead); Formal analysis (lead); Investigation (lead); Project administration (lead); Resources (lead); Software (lead); Validation (lead); Visualization (lead); Writing—original draft (lead); Writing—review & editing (lead). **Sanghyeon Lee**: Data curation (supporting). **Chihyeong Won**: Visualization (supporting). **Kyu Hyoung Lee**: Supervision (supporting); Validation (supporting). **Byeonggwan Kim**: Formal analysis (supporting). **Sungjoon Cho**: Formal analysis (supporting). **Taeyoon Lee**: Supervision (lead).

## Supporting information

Supplementary Material

## Data Availability

The data that support the findings of this study are available from the corresponding author upon reasonable request.
