# Supplementary material for: Stretchable Ag2Se Thermoelectric Fabric with Simple and Nonthermal Fabrication for Wearable Electronics
Source: Small Sci. 2024 Sep 1;4(11):2400230. doi: 10.1002/smsc.202400230 (PMC11935023; doi:10.1002/smsc.202400230)
Supplement: Supplementary file 1 — Supplementary Material [file SMSC-4-2400230-s001.zip › smsc202400230-sup-0001-SuppData-S1.pdf]

Supporting Information

Stretchable Ag_2Se Thermoelectric Fabric with Simple and Nonthermal Fabrication for Wearable Electronics

*Chaebeen Kwon, Sanghyeon Lee, Chihyeong Won, Kyu Hyoung Lee, Byeonggwan Kim, Sungjoon Cho and Taeyoon Lee**

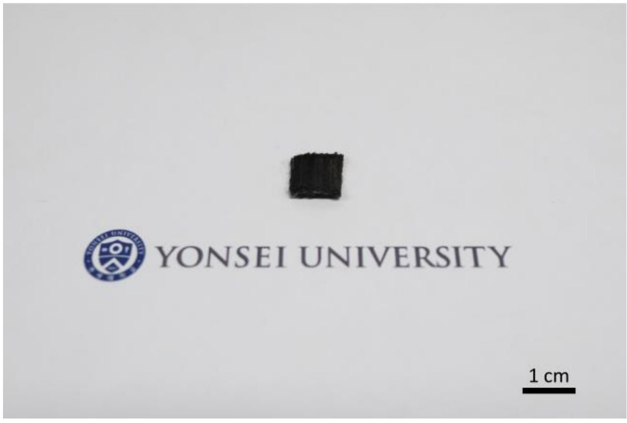

Figure S1. Optical image of Ag_2Se TE fabric.

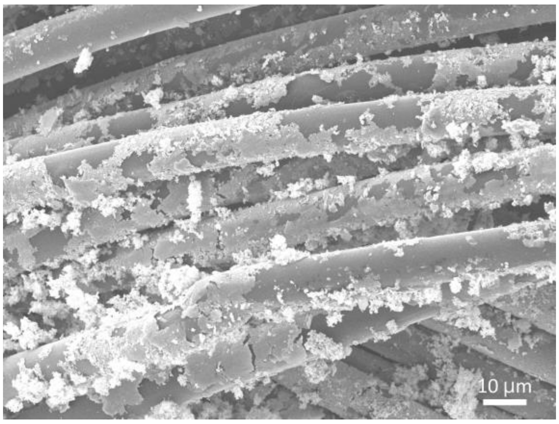

Figure S2. SEM images of Ag_2Se TE fabric with thermal treatment.

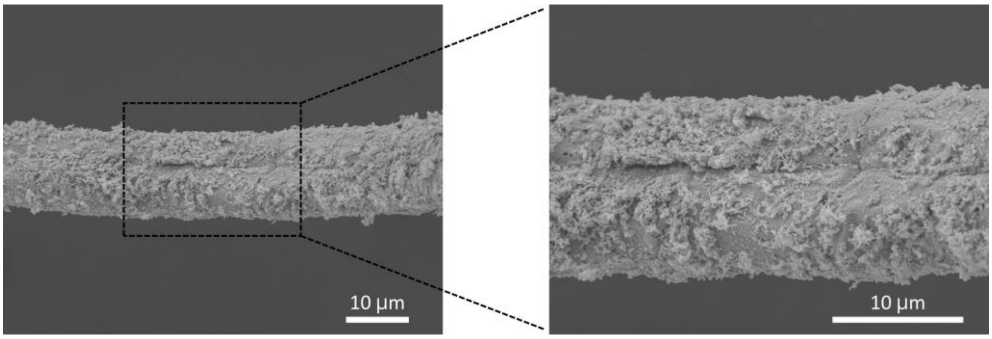

Figure S3. SEM images of single fiber from Ag_2Se TE fabric without thermal treatment.

Movie S1. Wearable haptic sensing glove using stretchable Ag_2Se TE fabric.

TE materials	From	Thermal treatment temperature (°C)	Fabrication time (h)	∣S∣ (μVK^{-1})	Stretchability (%)	Ref.
oleamine	fiber	60	> 13	64	~80	19
PEI@SWCNT	fiber	50	> 9	48	~37	20
PEDOT:PSS/EMIM:DCA	fiber	-	> 2	15	~30	21
$Ag_2Te_{0.6}S_{0.4}$	fiber	990	> 82	-	~21.2	22
Bi_2Te_3	fiber	950	> 2	150	-	23
Bi_2Te_3	fabric	80	> 5	83.79	~300	24
SWCNT	fiber	1100	-	120	-	28
SWCNT	fiber	130	> 2.5	44	~30	29
CNT	fabric	-	> 3.5	51	~250	30
Ag/Ag_2Se	fabric	200	> 1	90	~70	31
Ag_2Se	fabric	180	> 36	20	-	32
Ag_2Se	network	-	> 17.5	130	~100	33
Ag_2Se	fabric	-	< 1	26.98	~325	This work

Table S1. Comparison of this work with other TE fibers/fabrics.
